# Changes in the gut microbiota of mice orally exposed to methylimidazolium ionic liquids

**DOI:** 10.1371/journal.pone.0229745

**Published:** 2020-03-12

**Authors:** Gregory R. Young, Tarek M. Abdelghany, Alistair C. Leitch, Michael P. Dunn, Peter G. Blain, Clare Lanyon, Matthew C. Wright

**Affiliations:** 1 Faculty of Health and Life Sciences, Northumbria University, Newcastle upon Tyne, England, United Kingdom; 2 Department of Pharmacology and Toxicology, Faculty of Pharmacy, Cairo University, Cairo, Egypt; 3 Health Protection Research Unit, Institute of Cellular Medicine, Newcastle University, Newcastle Upon Tyne, England, United Kingdom; Medizinische Fakultat der RWTH Aachen, GERMANY

## Abstract

Ionic liquids are salts used in a variety of industrial processes, and being relatively non-volatile, are proposed as environmentally-friendly replacements for existing volatile liquids. Methylimidazolium ionic liquids resist complete degradation in the environment, likely because the imidazolium moiety does not exist naturally in biological systems. However, there is limited data available regarding their mammalian effects in vivo.

This study aimed to examine the effects of exposing mice separately to 2 different methylimidazolium ionic liquids (BMI and M8OI) through their addition to drinking water. Potential effects on key target organs–the liver and kidney–were examined, as well as the gut microbiome.

Adult male mice were exposed to drinking water containing ionic liquids at a concentration of 440 mg/L for 18 weeks prior to examination of tissues, serum, urine and the gut microbiome. Histopathology was performed on tissues and clinical chemistry on serum for biomarkers of hepatic and renal injury. Bacterial DNA was isolated from the gut contents and subjected to targeted 16S rRNA sequencing.

Mild hepatic and renal effects were limited to glycogen depletion and mild degenerative changes respectively. No hepatic or renal adverse effects were observed. In contrast, ionic liquid exposure altered gut microbial composition but not overall alpha diversity. Proportional abundance of *Lachnospiraceae*, *Clostridia* and *Coriobacteriaceae spp*. were significantly greater in ionic liquid-exposed mice, as were predicted KEGG functional pathways associated with xenobiotic and amino acid metabolism.

Exposure to ionic liquids via drinking water therefore resulted in marked changes in the gut microbiome in mice prior to any overt pathological effects in target organs. Ionic liquids may be an emerging risk to health through their potential effects on the gut microbiome, which is implicated in the causes and/or severity of an array of chronic disease in humans.

## Introduction

Ionic liquids are salts that are normally liquid at ambient temperature (typically below 100°C) and in some cases, are liquids at room temperature. These properties have resulted in them being proposed or used in a variety of industrial processes including processing of biomass; organic and inorganic materials syntheses; carbon dioxide capture; electrocatalysis; liquids for enzymes; lubricants and additives for conventional lubricants; battery electrolytes; use in fuel cells; use in separations and extractions for materials in the nuclear industry; separation agents and as pharmaceutical ingredients [1]. The low volatility of many ionic liquids in particular, may also be a driver for them being proposed as environmentally-friendly replacements for existing volatile solvents [1]. The term “ionic liquids” encompasses a diverse range of chemicals [1]. Five types of ionic liquid cations are currently mostly used:- ammonium, pyridinium, imidazolium, phosphonium and sulfonium [2].

Commercially widely-available methylimidazolium ionic liquids are a class of ionic liquids composed of a cationic methylimidazolium moiety with an alkyl chain increasing in length by 2 carbons (i.e. ethyl, 2C; butyl, 4C; hexyl, 6C….) and a variety of different anions (e.g. Cl-, Br-, tetrafluoroborate etc). In theory, a near limitless number of different methylimidazolium ionic liquids could be synthesised based on the many potential anions, but also on substitution at other positions around the imidazolium ring. However, based on registrations submitted to the European Chemicals Agency (ECHA), the total number of methylimidazolium ionic liquids used in the EU at present, is likely to be in the order of up to 60–70 [3]. Methylimidazolium-based ILs are most often proposed as solvents in a variety of industrial applications such as separations, catalysis and dissolution, with 1-ethyl-3-methylimidazolium acetate (likely used at high levels since the ECHA database indicates several variants manufactured at up to 100 tonnes/annum or manufacture levels are reported as confidential [3]). This methylimidazolium ionic liquid has shown outstanding performance in the fields of biomass dissolution and biopolymer processing [4].

From an environmental point of view, there are limited data to suggest that some methylimidazolium ionic liquids are resistant to rapid degradation. For example, according to the ECHA registration document for a 2C methylimidazolium ionic liquid, 1-ethyl-3-methyl-1H-imidazol-3-ium is not readily biodegradable (as determined using an OECD test guideline study using activated sludge). Microbiological metabolism does occur with methylimidazolium ionic liquids as the alkyl chain length increases however, cleavage of the imidazolium ring is not seen [5]. Their persistence in the environment may therefore be a cause for concern due to the presence of the imidazolium moiety, which does not exist naturally in biological systems.

Methylimidazolium ionic liquid toxicities have been most extensively examined in environmental toxicity tests. For example, for salts of 1-octyl-3-methylimidazolium chloride (M8OI), toxic effects have been demonstrated in *R*. *nigromaculata* frogs [6]; E. coli [7]; wheat [8,9]; green algae [10]; marine diatom *S*. *costatum* [11]; planarians *D*. *japonica* [12] and fish *P*. *dabryanus* [13].

Very limited data are publicly available regarding the potential toxicity of ionic liquids and methylimidazolium ionic liquids in particular, in mammalian systems. The NTP reviewed the literature on three 4C alkyl ionic liquids in 2004, including 1-butyl-3-methylimidazolium chloride (BMI) [14]. At that time, it was noted that information regarding acute, short-term/subchronic, or chronic exposure, synergistic/antagonistic effects, reproductive or teratological effects, carcinogenicity, genotoxicity or immunotoxicity were not available. To our knowledge, the database on methylimidazolium ionic liquid mammalian toxicity has not markedly increased except for a single study reporting acute toxic effects of the 8C alkyl ionic liquid 1-octyl-3-methylimidazolium bromide in mice over 24 hours after a single i.p. administration. Ten hours after administration, the authors report histopathological changes in the liver [15]. This observation has been followed by several in vitro studies in human liver cell lines showing that M8OI exposure leads to increased oxidative stress and cell death by an apoptotic mechanism(s) [16–19]. However, in our hands, the target organ for the toxic effects of M8OI after exposure by i.p. injection was the kidney [20].

Since ionic liquids are water-soluble and persist in the environment, the effects of extended exposure through drinking water is a potential route of exposure in man. Accordingly, the effects of exposing mice separately to 2 different methylimidazolium ionic liquids (BMI and M8OI) in their drinking water has been examined–to our knowledge for the first time–with a focus on the key target organs of liver and kidney. The gastrointestinal microbiota was examined given the apparent impact of the microbiota on a variety of chronic diseases [21,22]. M8OI was selected for study because the cation was recently detected at high levels in soils in close proximity to a landfill waste site [19] and is a potential hazard trigger for an autoimmune liver disease primary biliary cholangitis (PBC) [19,3]. BMI was included as this structurally-related ionic liquid is more widely used and is a more likely potential hazard trigger for PBC based on structural considerations [3].

This study explores the impact of oral exposure to ionic liquids in drinking water on murine gut physiology. We observe mild histological alterations contrasted with significant shifts in microbial communities.

## Materials & methods

### Materials

The chloride salts of BMI and M8OI were purchased from Sigma (Poole, UK) and were >99% and >97% pure respectively.

### Mice and experimental design

This study was performed under a licence by the UK Home Office with local Animal Welfare and Ethical Review Body (Newcastle University) approval. Adult C57Bl6 male mice (5 months of age) were purchased from Charles River and housed in the Comparative Biology Centre at Newcastle University. Mice (up to five per cage) were housed in Maxiseal 420 cm^2^ mouse cages (Arrowmight, Hereford, UK) in an enriched environment (nesting material, chew sticks and cardboard tubes) and were provided with food (RM3 Special Diet Services, UK) and water *ad libitum* in an air-conditioned environment on a 12 h light/dark cycle with regulated humidity (50% ± 10%) and temperature (23°C ± 1°C). Cages were randomly assigned to 3 groups and provided with drinking water *ad libitum* (control, 5 animals) or either drinking water containing BMI (10 animals) or M8OI (10 animals) at a concentration of 440 mg/L (prepared fresh every 4 weeks). Aliquots were tested by HPLC and both remained stable in drinking water for at least 4 weeks. No vehicle was required in these studies. One control mouse was culled during the study due to a handling error. There are no data available on potential exposure levels in man. A single dose was therefore chosen to reduce the number of animals used prior to any future dose-response study to determine a threshold for any effects. The dose chosen for this study was based on acute exposure via i.p. administration, as a dose likely to give no more than mild renal and hepatic effects. This route was chosen because the oral route is the most relevant route of exposure in man and relevant to test potential direct effects on the microbiota and liver. Based on a default factor for sub-chronic studies (0.15), it can be estimated that mice were exposed to 66 mg of BMI or M8OI/kg bw [23]. Mice were exposed for 18 weeks prior to cervical dislocation and removal of tissues, blood, and urine (by direct extraction from the bladder) for analyses. Gut contents were harvested by removing the intestinal tract and manually squeezing the excised caecal, proximal and mid colon contents in to sterile, DNA-free universals containing 2mLs sterile 1 x PBS (137 mM NaCl, 27 mM KCl, 100 mM phosphate pH 7.4). Single samples from each animal were snap frozen in dry ice then stored at -80°C until transportation on dry ice to Northumbria University for DNA isolation and processing. Due to the loss of one control mouse through a handling error, the gut contents of one stock animal was used as a replacement.

For examination of M8OI absorption, metabolism and excretion after oral exposure, mice (5 months of age) were randomly assigned to one of 4 dose groups:- control vehicle, 5 mg/kg bw, 20mg/kg bw and 40mg/kg bw and gavaged with M8OI in drinking water (8mL/kg bw). Control animals were gavaged with 8mL drinking water/kg bw. Mice were dosed at the beginning of the study and at 18 hours, prior to cervical dislocation at 24 hours and removal of tissues, blood, bile (by direct extraction from the gall bladder) and urine (by direct extraction from the bladder) for analyses.

### Pathology and clinical chemistry

Serum alanine aminotransferase (ALT) and alkaline phosphatase (ALP) activities and serum glucose and creatinine concentrations were determined as previously outlined [24]. Urinary total protein levels were determined using the Bradford protein assay. Urinary kidney injury molecule 1 (Kim1) levels were estimated by Western blotting as previously described [20]. Tissues were fixed in 4% formalin in 1xPBS, processed, embedded in wax and 4μm sections stained with haematoxylin and eosin (H&E) or sirius red (followed by haematoxylin) essentially as previously outlined [24]. Liver sections were also periodic acid stained (PAS) with or without prior treatment with diastase essentially as described [20]. Sections were also subjected to immunohistochemical analyses for vimentin, α-smooth muscle actin and Kim1 as previously outlined [25].

### Bacterial DNA isolation from murine stool

Processing order of gut content samples was randomised to reduce confounding effects. Prior to DNA isolation, all samples were defrosted at room temperature, homogenised by vortexing, then centrifuged at 6000 x g for 25 minutes to pellet biological material. Supernatants were discarded and 250 mg of each pellet was transferred to individual bead tubes of QIAGEN PowerLyzer PowerSoil DNA Isolation kit (Hilden, DE). DNA isolation was performed as per manufacturer’s instructions with an extended bead beating step of 25 minutes. All extractions were performed alongside 2 buffer (consisting of only 1 x PBS used to store samples) and 1 kit (consisting of only kit reagents) control.

### 16S rRNA sequencing and processing

The Schloss SOP [26], was followed to prepare libraries of isolated bacterial DNA for multiplexed, targeted sequencing of the V4 region of the 16S rRNA gene on the Illumina MiSeq (CA, USA), with V2 chemistry (2 x 250 bp reads), using primers 515F and 806R [27]. A sequencing negative control was also included consisting of sterile DNA-free H_2_O.

Forward and reverse reads were merged, quality filtered, trimmed, clustered in to de novo operational taxonomic units (OTUs), and assigned taxonomy based on the SILVA database [28], in Mothur according to the MiSeq SOP [29]. Briefly, all reads with phred score less than q30, ambiguous bases and / or greater than 275 bp in length were culled. Any reads not aligned to the SILVA database were removed along with non-bacterial sequences and chimeric sequences as defined by standard uchime operating parameters [30]. For an overview of the microbial ecology terms used and data on raw sequence reads and processing, see S1 and S2 Materials respectively.

### M8OI and metabolite determination in biological samples

Biological samples (serum, bile and urine) were analysed for the presence of M8OI and its major metabolites—HO8IM and HOOC7IM—essentially as previously described [19] using non targeted data independent LC-HR-MS/MS using a TripleTOF 5600 high-resolution quadrupole time-of-flight (TOF) mass spectrometer (Sciex) equipped with a DuoSpray ion source operated in positive electrospray mode, coupled to an Eksigent Nano LC 420 system. M8OI and metabolites were quantified by standard multiple reaction monitoring (MRM) techniques using a Q-Trap 5500 hybrid linear ion trap/triple quadrupole mass spectrometer (Sciex) coupled to a Shimadzu Prominence liquid chromatograph. Analyst version 1.6.2 and MultiQuant version 2.0 (Sciex) were used for instrument control/data acquisition and quantitative analysis respectively.

### Statistical analyses

For comparing animal sample endpoints between two groups, an unpaired Students t-test was carried out and significance assumed where p<0.05. For comparison of multiple groups, ANOVA was used and, where significant, differences between exposure groups were determined using the Bonferroni-Holm method. Where p<0.05, a significant difference was assumed.

Analysis of microbial communities was performed in R [31], utilising phyloseq [32], vegan [33], and pairwiseAdonis [34] packages. Data were visualised with the ggplot2 [35], and ggbiplot [36] packages. To normalise variable sequencing depth per sample, raw counts of OTUs were expressed as relative abundance per sample. Comparison of negative control and test communities were performed by Adonis PERMANOVA. Fisher’s alpha index was calculated to determine alpha diversity within samples while Bray-Curtis dissimilarity was calculated to determine beta diversity between samples. Kruskall-Wallis rank sum test was invoked to assess significant differences between continuous variables such as relative abundance of individual genera or OTUs between exposure groups. Pairwise Mann-Whitney Wilcoxon test was utilised to cross-compare multiple groups of continuous variables such as alpha diversity between exposure groups.

Complete linkage clustering based on beta diversity was validated by gap statistic and used to differentiate samples in to community state types (CSTs) as previously described [37]. ANOSIM was employed to identify significant dissimilarity between CSTs while pairwise PERMANOVA further identified significant dissimilarity between each CST. Bacterial OTU dominance was calculated using inverse Simpson diversity index. Relationships between xenobiotic exposure cohort and CST membership were assessed using Fisher exact test.

Metagenomes were predicted using PICRUSt [38] based on bacterial taxonomies assigned by closed reference alignment of raw sequence reads to the Greengenes database [39]. Differential KEGG pathways between predicted metagenomes were identified by linear discriminant analysis effect size (LefSe, [40]), after normalisation by converting to relative abundance.

Wherever necessary, calculated p values were corrected for multiple hypothesis testing by false discovery rate (FDR), or Bonferroni correction.

## Results

### Hepatic and renal effects of ionic liquid exposure

Prior to the study, drinking water consumption by mice was monitored and no changes in consumption were observed once mice were placed on drinking water containing ionic liquids. No overt adverse effects were observed throughout the 18-week exposure period based on general observations in behaviour and visual observations. One control mouse was culled during the study due to a handling error.

Table 1 indicates that ionic liquid exposures did not have a statistically significant effect on terminal body weights. There were no statistically significant effects on relative liver weights or evidence for overt liver injury based on changes in serum ALT or ALP activities. No significant effects were seen on serum glucose or liver glycogen levels (S1 Table). On examination for evidence of renal injury, the only marker statistically significantly raised relative to control was urinary Kim1 protein in mice exposed to BMI. However, a statistically significant change in other renal injury markers (serum creatinine, urinary protein) in BMI treated animals was not detected (Table 1).

**Table 1:** Effect of 18 week exposure to BMI or M8OI in drinking water to male mice.

	*Control*		*BMI*		*M8OI*	
*Endpoints*	Mean	SD	Mean	SD	Mean	SD
*General*						
Body weight (g)	36.70	3.59	34.10	3.49	33.90	2.49
*Hepatic*						
Relative liver weight (% body weight)	4.50	0.48	4.30	0.48	4.60	0.63
Serum ALT (units/L)	36.10	23.70	35.41	9.27	39.50	19.87
Serum ALP (units/L)	64.00	4.30	66.00	18.20	69.00	22.60
*Renal*						
Serum creatinine (units/L)	22.00	4.00	21.00	8.30	27.00	10.10
Urinary protein (mg/mL)	0.66	0.24	0.75	0.40	1.30	0.50
Urinary Kim1 protein (units/7.5μl)	1.70	0.52	15.00*	8.90	6.40	3.13

*Significantly different (two tailed) from control using a one-way ANOVA followed by bonferroni post hoc test.

To determine whether there were any potential localised hepatic and renal effects of ionic liquids, organs were fixed and processed for histological and immunohistological examination.

**Fig 1:**
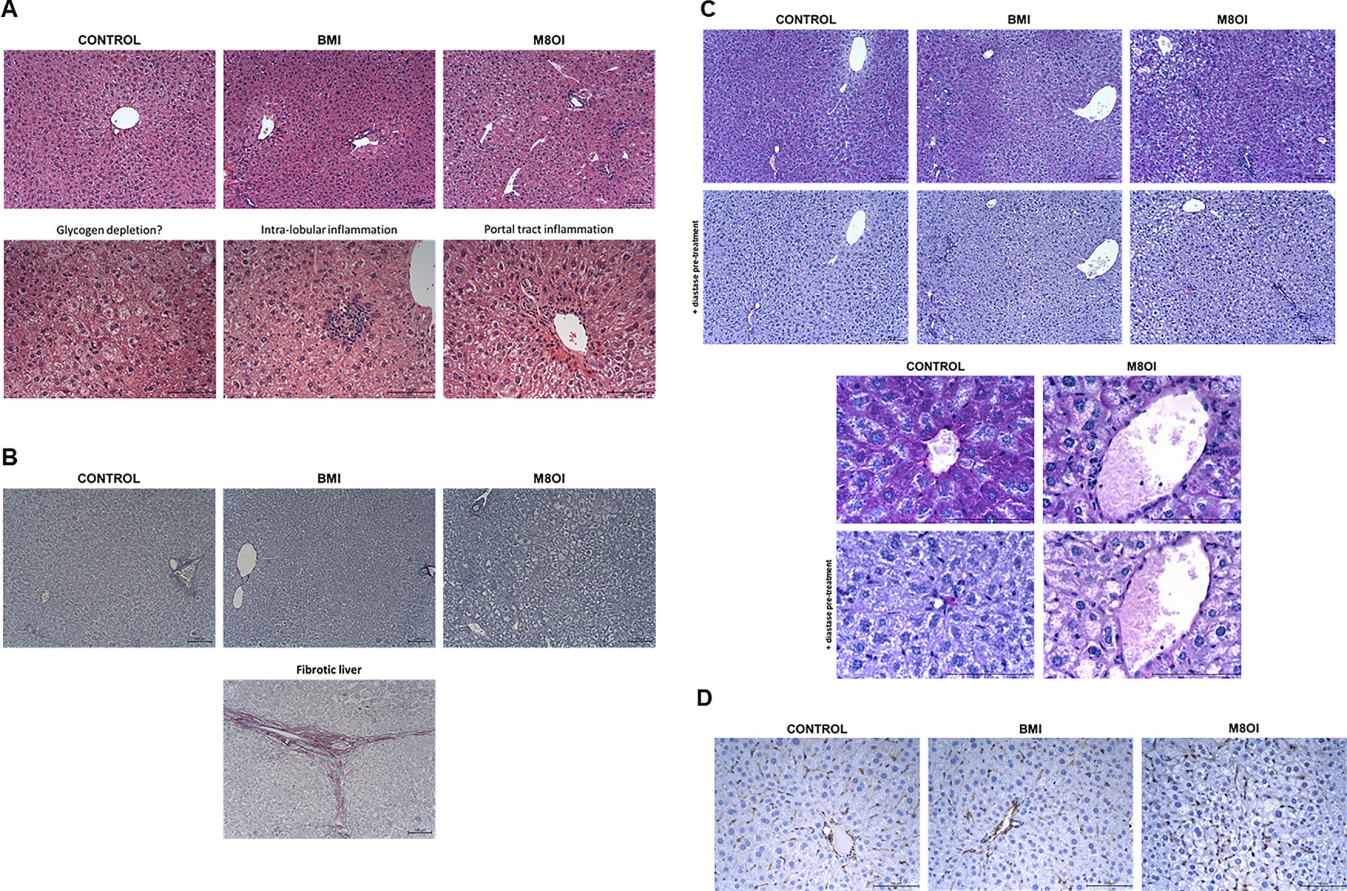
Histopathological effects of BMI or M8OI exposure in the liver. **A**, upper panels, typical low powered views of liver sections stained with H&E from the indicated treatment groups. Lower panels, high powered views of some changes:- hypoeosinophilia (left panel) which was more widespread in M8OI-treated mice but also present in other groups and intra-lobular and portal tract inflammation, both observed to a near similar extent in all groups. **B**, typical low powered views of liver sections stained with Sirius red (staining collagen fibres red), followed by haematoxylin from the indicated treatment groups. Lower panel, positive control–liver section from a donor liver organ with cirrhosis. **C**, typical low powered views (upper panels) and high powered views (lower panels) of liver sections stained with PAS from the indicated treatment groups, with where indicated, diastase treatment. **D**, typical low powered views of liver sections immunostained for vimentin from the indicated treatment groups. Size bar = 100μm throughout.

Examination of H&E-stained liver sections supports the clinical chemistry endpoints (ALT, ALP), with no evidence for any on-going hepatocyte necrosis (Fig 1A) and no evidence for unresolved chronic injury based on an absence of fibrosis (Fig 1B). No evidence for an increase in liver cell apoptosis (based on active caspase 3 expression in liver sections) was observed (data not included).

Patchy areas of hypoeosinophilia suggestive of protein breakdown were observed and were more common and widespread in ionic liquid-treated mice (Fig 1A), see also S2 Table for individual scoring data. Patchy and variable degrees of portal tract and intra-lobular inflammation (Fig 1A) were, however, also evident. This background pathology is common (though also variable within the same liver and between animals) in mice and was not observed to be significantly markedly different in mice exposed to ionic liquids (S2 Table).

**Fig 2:**
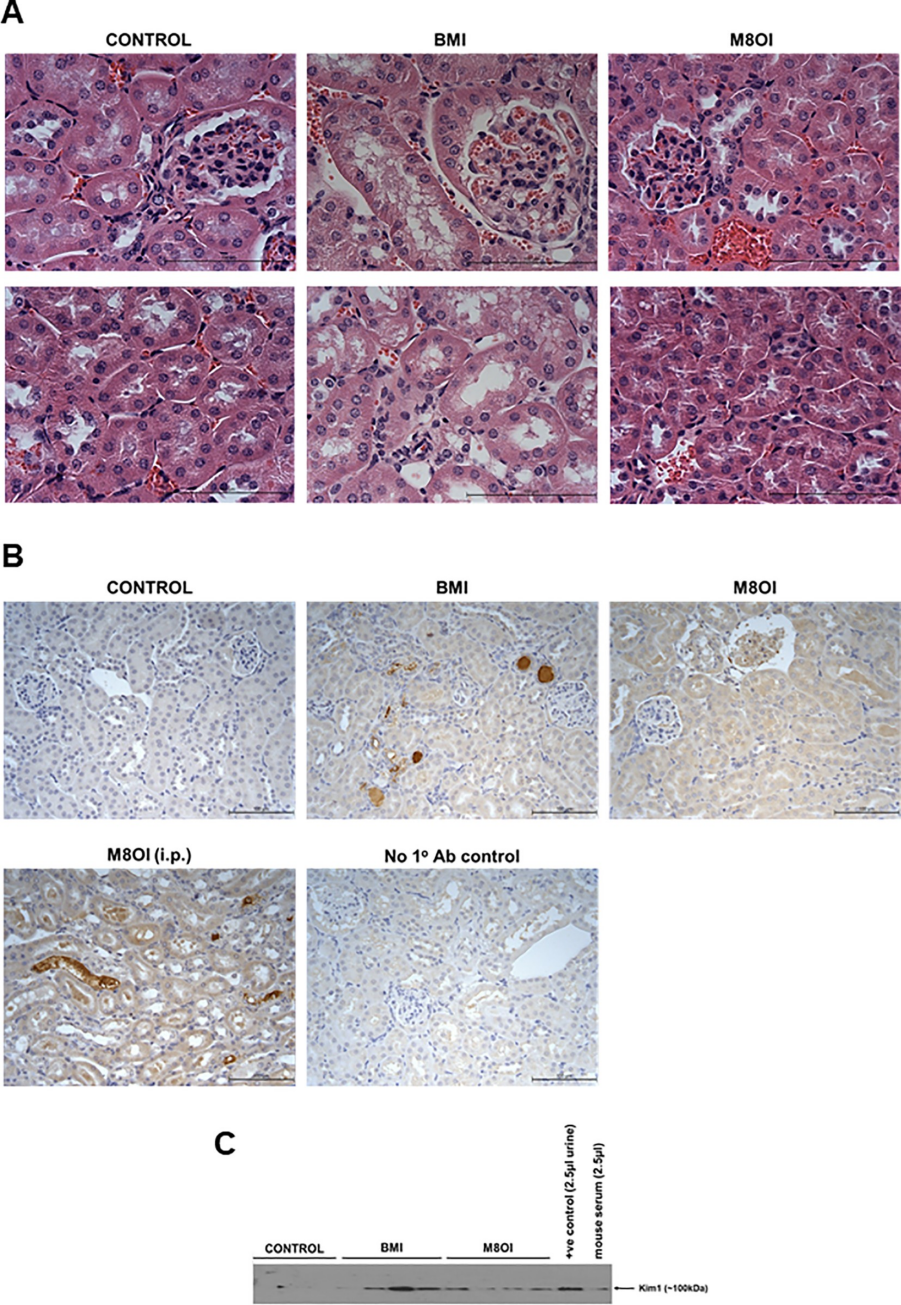
Histopathological effects of BMI or M8OI exposure in the kidney. **A**, typical views of kidney sections stained with H&E from the indicated treatment groups. Upper panels showing glomeruli, lower panels, tubules. **B**, typical views of kidney sections immunostained for kim1 from the indicated treatment groups. M8OI (i.p.) kidney section from a mouse administered M8OI via intraperitoneal injection; No 1^o^ Ab, section treated identically without the addition of primary (i.e. kim1) antibody. Size bar = 100μm throughout. **C**, Western blot for kim1 in urine from individual mice (7.5μl/lane). +ve control, urine from a mouse administered M8OI via intraperitoneal injection [20].

To determine whether glycogen depletion was in part associated with hypoeosinophilia evident in H&E-stained liver sections, glycogen was specifically examined using PAS staining (with and without prior glycogen digestion with diastase). Based on these data, the only consistent hepatic effect observed to be markedly different between control and ionic liquid-treated mice was a variably patchy depletion of glycogen (Fig 1C), with increased association of vimentin-expressing fibroblasts (Fig 1D). The effect was observed to be most marked in M8OI-treated mice yet was also variable in that some mice were resistant. The patchy nature of the effect was also limited such that total glycogen levels were not significantly reduced (S1 Table) in contrast to a complete depletion after i.p. administration of M8OI (2 x 10mg/kg body weight split over a 24 hour period) [20]. An examination of H&E-stained kidney sections indicated focal and mild degeneration in a limited number of ionic liquid-exposed mice. For individual scoring data, see S3 Table. The necrotic changes were characterised by hydropic degeneration and desquamation of cells from tubules (Fig 2A). Fig 2B indicates that there was a moderate increase in kidney kim1 tissue expression relative to control in ionic liquid-treated mice, with BMI-treated mice showing a higher incidence for tubular kim1 levels compared to M8OI-treated mice, which is supported by the higher levels of urinary kim1 protein from selected mice (Fig 2C). However, Fig 2B indicates that these effects in orally exposed BMI- or M8OI-treated mice were less severe than the effects seen from an i.p. administration of M8OI (2 x 10mg/kg body weight split over a 24-hour period).

### Oral exposure to M8OI results in absorption followed by renal and biliary excretion of M8OI and its metabolites HO8IM and HOOC7IM

**Fig 3:**
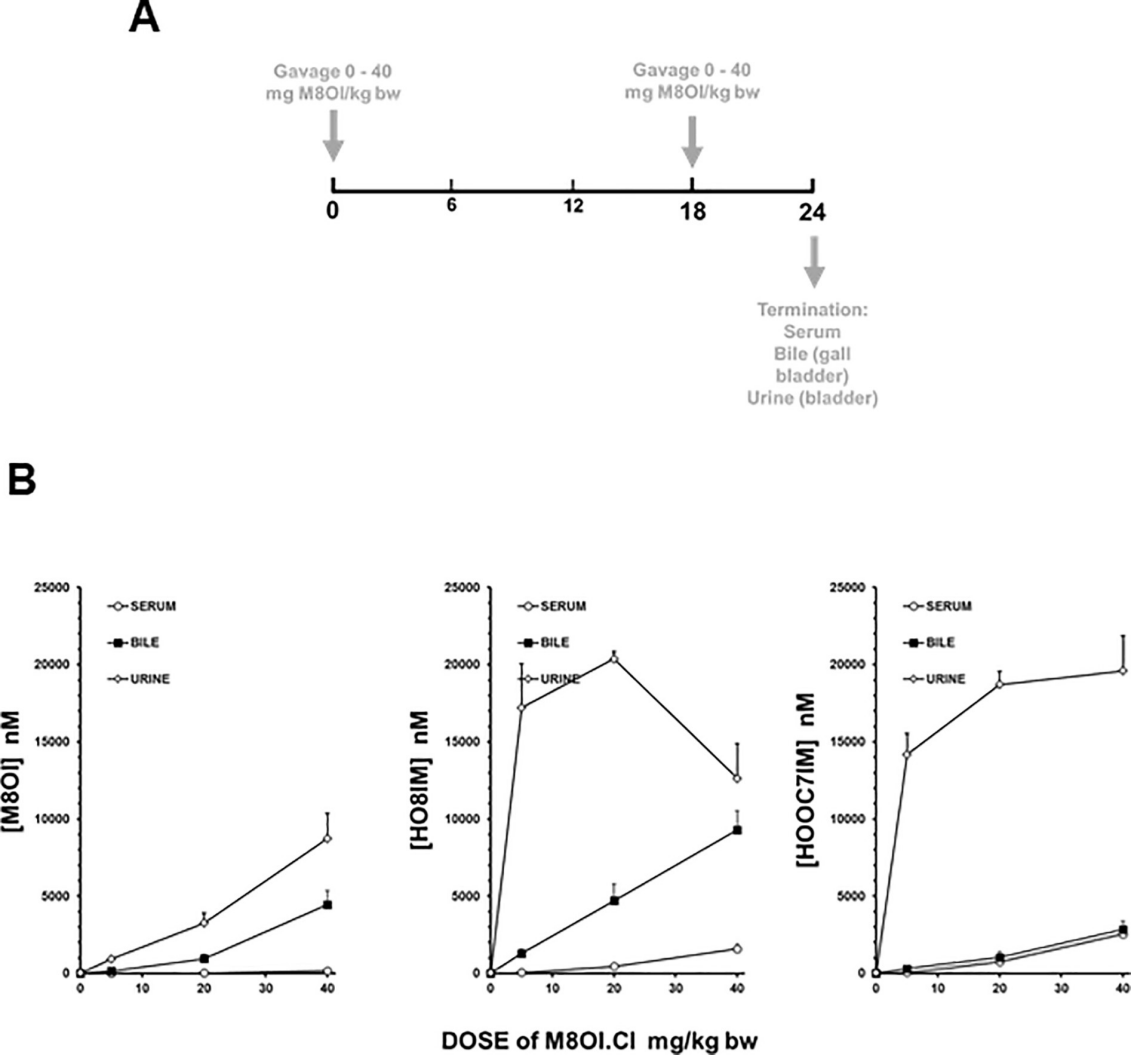
The disposition of M8OI in the mouse after oral gavage. **A**, schematic diagram of dosing regimen used. B, serum, bile and urine concentrations of M8OI or the hydroxylated (HO8IM) and carboxylated (HOOC7IM) metabolites at termination. Data are the mean and SD of 5 individual animal serum samples at any one timepoint. Bile and urines data are the mean and SD of between 3 and 5 individual animal samples. Note, in some cases, sufficient urine or bile was not obtained from an animal.

The fate of M8OI was examined after oral administration to mice by gavage as outlined in the methods section (and illustrated in Fig 3A) using an established assay [19] and the levels of M8OI and its metabolites determined in serum, bile and urine at time of sacrifice. Fig 3B demonstrates that M8OI was absorbed from the gastrointestinal tract and systemically distributed since it appears at an increasing concentration in the urine with increasing dose. However, M8OI is likely rapidly cleared from the systemic circulation since it is barely detectable in the serum at termination at any dose. The appearance of M8OI in an increasing concentration in both urine and bile with dose indicates both biliary and renal excretion, with renal clearance likely playing the major role (Fig 3B). In contrast to M8OI, the metabolites shown to be produced by the human liver [19] are readily detectable in the serum with both metabolites also being excreted via the bile and urine (Fig 3B). Renal excretion of the metabolites appeared to become saturated at the high dose level, particularly for HO8IM (Fig 3B), which may lead to increased biliary excretion.

In the absence of an assay for BMI, its absorption and bioavailability were predicted *in silico* along with a range of other structurally-related methylimidazolium ionic liquids (including M8OI). S1 Fig shows that the *in silico* prediction for oral absorption and bioavailability of M8OI is supported by the data obtained in orally-dosed M8OI in mice (Fig 3) in that there is a minor but significant absorption but low bioavailability of M8OI (due to metabolism and excretion). BMI is predicted to show a greater degree of absorption from the gastrointestinal tract and to have higher bioavailability than M8OI (S1 Fig).

These data suggest that exposing mice for 18 weeks to BMI or M8OI in their drinking water results in absorption and both hepatic and systemic exposure to the ionic liquids and/or their metabolites based on M8OI and metabolite determinations in biological fluids, *in silico* predictions of absorption and bioavailability, hepatic glycogen depletion and renal degenerative changes. However, these effects were mild and limited in comparison to the effects (previously observed) seen after an acute systemic exposure to M8OI (via an i.p. administration of M8OI; 2 x 10mg/kg body weight split over a 24-hour period). On this basis, no hepatic or renal adverse effects were observed when exposing mice to the ionic liquids in drinking water at the doses employed.

### Exposure to ionic liquids impacts gut microbial composition

To determine whether there had been any changes to the gut microbiota in mice exposed to ionic liquids, bacterial DNA was isolated from the gut contents and subjected to targeted 16S rRNA gene sequencing and processing as outlined in the Methods section. A total of 1.71 x 10^6^ total 16S rRNA gene sequence reads (mean = 6.34 x 10^4^, SD = 3.74 x 10^4^), passing quality filter were observed across n = 27 samples, including all negative controls (S2 Fig). Negative control microbiota were compared with sample compositions, showing significant dissimilarity (p = 0.004, R_2_ = 0.30 [adonis PERMANOVA]) (S3 Fig). Negative controls (n = 4), were omitted from subsequent analyses and the dominant OTU (classified as *Escherichia/Shigella*) present in negative controls was removed from the dataset. The remaining 25 samples yielded 1.71 x 10^6^ total 16S rRNA gene sequence reads.

**Fig 4:**
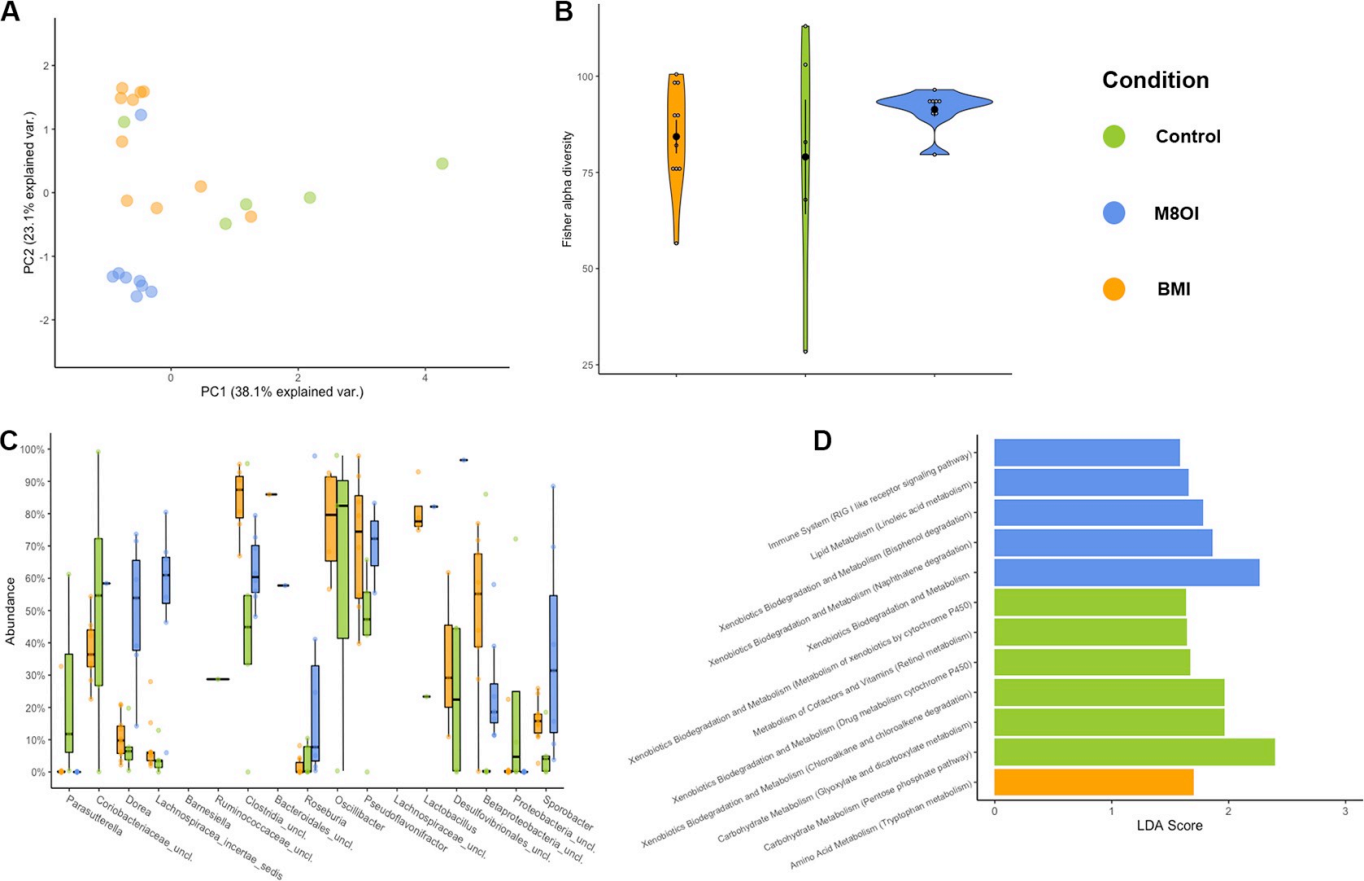
Impact of ionic liquid exposure on gut microbiota composition. **A**, Panels illustrate beta diversity, expressed as Bray-Curtis dissimilarity. **B**, alpha diversity, expressed as fisher-alpha diversity. **C**, significantly differential abundant microbial genera identified. **D**, KEGG functional pathways identified between exposure groups. Each point in panels a–c represents an individual sample. Points are coloured by exposure condition (M8OI = blue; BMI = orange; Control = green). Ordination in panel A describes 61% of the total dissimilarity between samples. KEGG pathways in panel D were identified by PiCrust metagenome predictions based on taxonomic composition.

Gut microbial community composition was different in mice exposed to the ionic liquids compared to controls (Fig 4A) (p = 0.001, R_2_ = 0.26 [Adonis PERMANOVA]). Exposure to ionic liquids significantly reduced the beta diversity (unweighted Bray-Curtis dissimilarity) observed within exposure groups (Fig 4A). Thus, exposure to ionic liquids had a consistent effect on microbiota composition, reducing the within group community variation. These results were particularly evident in M8OI exposed mice. Indeed, the community composition of mice exposed to M8OI was significantly different to both BMI and control subjects (Table 2).

**Table 2:** Comparison of microbial beta diversity of gut contents from each cohort.

*Cohort*	*BMI*	*Control*
*BMI*	-	0.021*
*M8OI*	0.003*	0.006*

*Statistically significant difference in beta diversities (Bray-Curtis) between pairwise cohort comparison (pairwise PERMANOVA with Bonferroni correction).

Further evidence of the consistent effects and reduced variation between microbiota of M8OI exposed mice compared to both BMI and control groups is highlighted in Fig 4B. Despite no observed significant differences in alpha diversity (Fisher’s alpha), between exposure groups the within group variation observed for M8OI exposed mice was much lower than in BMI exposed and control mice.

### Ionic liquid exposure enriched specific taxonomic and functional repertoires

To identify taxa responsible for the consistent changes and significant differences in community composition observed in the three ionic liquid exposure groups, relative abundance of merged genera (agglomerated using ‘tax_glom’ function in phyloseq R package [30]), were compared between controls and ionic liquid exposed mice.

Significant differences in relative abundance of 13 different genera were observed between control and ionic liquid exposed mice (Fig 4C, Table 3). Significantly greater relative abundance of *Parasutterella*, a common core component of the murine microbiota [41], was observed in control mice than both ionic liquid exposed groups. Lower levels of *Parasutterella* in ionic liquid exposed mice was countered with greater relative abundance of genera belonging to the *Lachnospiraceae*.

**Table 3:** Seventeen most significantly differential genera between gut microbiota of each treatment group.

	*Control*		*BMI*		*M8OI*			
*Genus*	Mean Abundance	SD	Mean Abundance	SD	Mean Abundance	SD	P val	FDR adjusted P val
*Parasutterella*	13.07	23.86	1.55	1.07	3.42	2.74	0.00	0.02
*Coriobacteriaceae_uncl*.	0.60	0.43	0.62	0.39	1.15	0.43	0.00	0.02
*Dorea*	0.39	0.61	0.38	0.64	1.37	0.91	0.00	0.02
*Lachnospiracea_incertae_sedis*	0.09	0.08	0.07	0.04	0.55	0.21	0.00	0.02
*Barnesiella*	1.65	2.03	3.07	2.08	1.52	1.13	0.00	0.02
*Ruminococcaceae_uncl*.	0.42	0.25	0.02	0.03	0.42	0.42	0.00	0.02
*Clostridia_uncl*.	0.52	0.38	0.85	0.33	0.92	0.43	0.00	0.02
*Bacteroidales_uncl*.	3.06	2.87	3.44	1.16	2.73	1.56	0.01	0.03
*Roseburia*	0.31	0.30	0.53	0.48	0.98	0.62	0.01	0.03
*Oscillibacter*	1.10	0.76	1.55	1.07	2.18	1.03	0.01	0.03
*Pseudoflavonifractor*	1.42	2.19	0.51	0.40	0.87	0.80	0.01	0.04
*Lachnospiraceae_uncl*	14.50	23.09	31.71	21.29	22.22	14.86	0.01	0.04
*Lactobacillus*	1.15	0.67	1.80	1.15	3.87	2.80	0.01	0.04
*Desulfovibrionales_uncl*.	0.04	0.05	0.15	0.18	0.62	0.39	0.02	0.07
*Betaproteobacteria_uncl*	0.34	0.21	0.90	0.26	1.02	0.46	0.03	0.07
*Proteobacteria_uncl*.	1.37	2.22	0.18	0.30	0.56	0.80	0.03	0.09
*Sporobacter*	0.06	0.08	0.16	0.07	0.51	0.51	0.04	0.09

Dotted line indicates threshold of genera present at significantly different relative abundance between cohorts (Kruskall-Wallis rank sum test) following FDR correction. Uncl = unclassified; SD = standard deviation; FDR = False Discovery Rate; val = value.

*Coriobacteriaceae spp*. were the most significant differentially abundant bacterial feature identified between subjects exposed to M8OI and controls (Fig 4C, Table 3), exhibiting a 1.9 fold change in mean abundance. This is of particular interest as the genera within the *Coriobacteriaceae* have previously been associated with xenobiotic metabolising potential [42,43]. This is explored further in this population via predicted metagenomes. Furthermore, Bendtsen and colleagues observed increased abundance of *Coriobacteriaceae* in mice exposed to increased stress levels in the living environment [44]. Psychological stress has previously been linked with inflammatory processes [45].

Significantly greater relative abundance of several members of the *Lachnospiraceae* family: *Dorea* (fc = 3.52)*; Lachnospiraceae_incertae_sedis* (fc = 6.35); *and Roseburia* (fc = 3.15), were observed in M8OI exposed mice compared to controls. This suggests the family may possess a fitness advantage over other bacteria following exposure to the ionic liquids used in this study. In addition, the unclassified *Lachnospiraceae* genus exhibited a 2.18 fold increase in mean relative abundance between BMI exposed and control mice.

Other genera associated with exposure to M8OI included an unclassified *Clostridia*, *Oscillibacter* and *Lactobacillus*. Genera associated with exposure to BMI included *Barnesiella* and *Roseburia* The greatest significant fold change in mean abundance was observed in *Ruminococcaceae*, which exhibited a 25.64 fold decrease following BMI exposure though was never present at greater than 1% of the total observed genera within any sample.

Supplementary to changes in individual bacterial genera, predicted metagenomes of communities exposed to each chemical illustrated altered functional capacities (Fig 4D). Significant changes in several KEGG pathways associated with metabolic potential were identified by linear discriminant analysis effect size [40]. Specifically, mice exposed to M8OI harboured microbiomes with significantly greater relative abundance of KEGG pathways associated with xenobiotic metabolism, particularly those for bisphenol and naphthalene metabolism. In contrast, mice exposed to BMI showed greater relative abundance of KEGG pathways associated with metabolism of amino acids such as tryptophan. Furthermore, mice exposed to either xenobiotic, exhibited lower relative abundance of KEGG pathways associated with metabolism of retinol and lipids, unsaturated fatty acid biosynthesis and cytochrome p450 activity.

### Gut microbiota cluster by ionic liquid exposure groups

**Fig 5:**
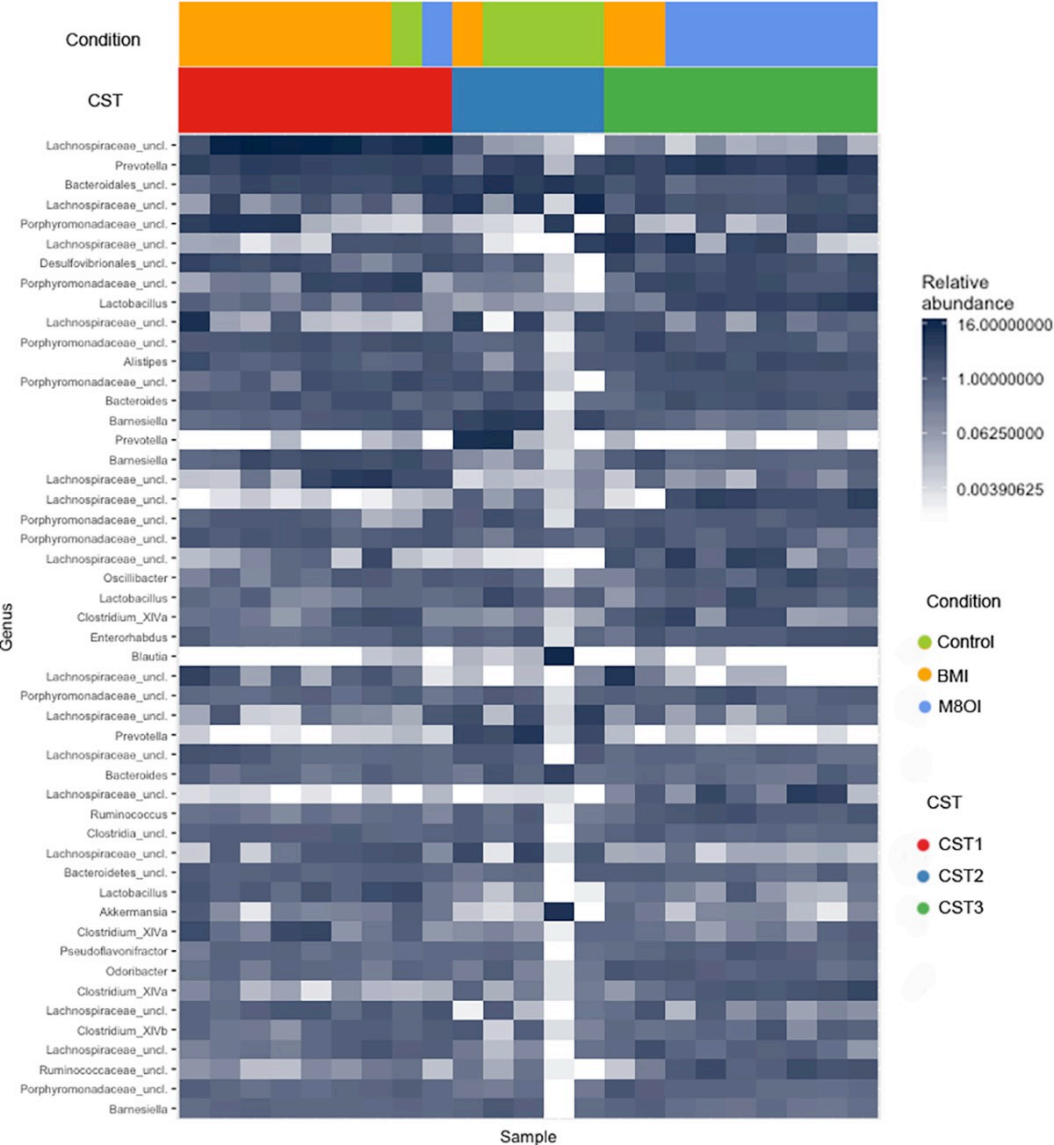
Complete linkage hierarchical clustering identifies three distinct community state types based on relative abundance of bacterial OTUs. Membership of CSTs shows significant association with ionic liquid exposure group and is also linked with sample alpha diversity. Heatmap shows relative abundance of the top 50 most abundant bacterial OTUs. Each tile represents the abundance of a specific OTU (in rows), in the given sample (in columns).

Samples formed three distinct clusters, referred to as community state types (CSTs), when beta diversity (Bray-Curtis dissimilarity), was compared by complete linkage hierarchical clustering (Fig 5, S4 Fig, S4 Table). Membership of each distinct CST was significantly associated with an individual ionic liquid exposure group (p < 0.001 [Fisher exact test]). Specifically, mice exposed to BMI were significantly associated with CST1, M8OI exposed mice were more likely to be members of CST3 and control mice dominated CST2 (Fig 5). Significant differences were observed in OTU evenness (Inverse Simpson diversity), between CSTs. CSTs associated with ionic liquid exposure exhibited significantly lower OTU evenness than CST3, associated with control mice (CST1 vs CST3 p = 0.037; CST2 vs CST3 p = 0.012 [Bonferonni corrected pairwise Mann-Whitney Wilcoxon test]). These results are as expected from the differential genera analyses (Fig 4C, Table 3), in which greater evenness was illustrated in control mice by an absence of any dominant genera, compared to both ionic liquid exposed groups.

## Discussion

This study is the first–to our knowledge–to examine exposing animals, orally and over an extended period of time, to ionic liquids.

To date, only two studies have examined the effects of methylimidazolium ionic liquids in vivo. Both studies were performed in mice with M8OI over a 24-hour period. The first study used the Br^-^ salt of M8OI and reported marked hepatic effects within 16 hours after a single i.p. injection [15]. A more recent study from this laboratory used the same route of exposure but employed lower split doses and the Cl^-^ salt (to avoid any complications from the anion), as used in these studies [20]. This latter study did not replicate the striking hepatic effects seen with the Br^-^ salt although this may be due to differences in the salt used and the doses employed. However, as observed in the current study, administration of the Cl^-^ salt of M8OI did not result in necrosis of hepatocytes in the liver based on both histopathological examination and an absence of any increase in serum ALT or ALP [20]. However, i.p. administration of M8OI did result in a significant dose-dependent loss of hepatic glycogen and a mild but significant increase in portal tract inflammatory recruitment and/or fibroblastic proliferation accompanied by a focal fibrotic change [20]. The kidney was found to be the organ most affected (no effects were seen in the several other organs: brain, heart and pancreas) after i.p. administration of M8OI, as it resulted in focal and mild degeneration to multifocal and moderate generation with a general trend for an increase in severity with increased dose [20]. The relatively milder hepatic and renal effects of M8OI seen in the current oral study, compared to an acute i.p. exposure, suggests that there may be limitations in the absorption of M8OI from the gastrointestinal tract and/or a threshold to any adverse systemic effects to M8OI administered as a bolus. Examining the disposition of M8OI after oral bolus administration in this study confirms that M8OI is absorbed to some extent since M8OI and its known metabolites appear in the bile and urine. However, the low serum levels of M8OI suggest it may be subject to rapid excretion and low bioavailability, which may account for its limited systemic-dependent effects. No studies with BMI have been performed in animals and it is interesting to note that this ionic liquid–which shows less toxicity to cells in vitro [In general, the longer the alkyl chain in methylimidazolium ionic liquids, the more toxic is the ionic liquid (Abdelghany *et al*, manuscript in submission)]–has resulted in more severe renal effects than M8OI. This latter effect may be associated with its higher absorption and bioavailability, as predicted in silico in this study.

Although the effects of exposing mice to ionic liquids in their drinking water had minimal effect on the target organs, the effects on the gut microbiota were significant. Specifically, exposure to M8OI had a profound impact on caecal microbiota beta-diversity compared to both controls and BMI exposed mice. Indeed, clustering of samples based on beta-diversity identified 3 distinct clusters, each associated with a single exposure group. Proportional abundances of several taxa were different in mice exposed to ionic liquids, along with KEGG pathways indicated from predicted metagenomes.

Exposure to M8OI resulted in significantly greater relative abundance of *Coribacteriaceae* and *Lachnospiraceae* as well as KEGG pathways associated with metabolism of xenobiotics. Metabolic functional capacity was predicted based on community membership rather than directly measured in this study. Nonetheless, *Coriobacteriaceae* have previously been linked with xenobiotic degrading potential [39,40]. Expansion of the *Lachnospiraceae* family may be a direct result of the reduction in *Parasutterella* and *Ruminococcacea* as both *Lachnospiraceae* and *Ruminococcacea* share a considerable overlap in functional repertoires including metabolism of host-derived glycans via the acrylate and propionate pathways [46,47]. The greater proportional abundance of both *Coriobacteriaceae* and *Lachnospiraceae* along with their associated KEGG pathways following exposure to M8OI suggests metabolic plasticity within these taxa may confer a fitness advantage during exposure to xenobiotics. It is, however, important to add that the increased abundances of both these KEGG pathways and bacterial families exhibited here are purely observational.

BMI exposed mice showed greater proportional abundance of *Clostridia* and of KEGG pathways associated with amino acid metabolism than control mice. The potential of *Clostridia* to metabolise aryl amino acids has been previously described [48]. Indeed, *Clostridia* are the most common fermenters of amino acids found in the gut [49]. Excessive breakdown of proteins by the gut microbiota can lead to build up of toxic end products such as phenols, ammonia, amines and hydrogen sulphide [49,50]. This is of particular interest due to the more severe renal impacts of BMI than M8OI in this cohort.

Significantly greater proportional abundance of xenobiotic metabolising potential identified in M8OI than BMI exposed mice may suggest the former is more readily degraded by the gut microbiota. This could explain the reduced impact of orally delivered M8OI in this study, compared to previous work [20], in which M8OI was delivered via i.p. injection. Moreover, this may explain the difference in observed renal damage between M8OI and BMI. The differences in degrading potential may be linked to length of the alkyl chains where the impact of steric interference by the imidazole ring is reduced as the alkyl chain extends from 4C in BMI to 8C in M8OI. Degradation of M8OI by the gut microbiota may also explain the lower bioavailability of M8OI compared to BMI identified by our *in silico* analyses.

Primary biliary cholangitis (PBC), is a progressive liver disease manifesting in non-obstructive damage to the small bile ducts causing cholestasis and, in late-stage disease, cirrhosis, sometimes necessitating transplant [51]. Exact aetiology remains enigmatic though it is widely considered an autoimmune condition due to presence of anti-mitochondrial antibodies (AMAs) to the lipoyl domains of autoreactive mitochondrial proteins [52–55]. There is a clear genetic predisposition to developing PBC, with greater pairwise concordance rates between monozygotic twins (0.63), than among other autoimmune conditions [56]. Geographic clusters of PBC in non-related individuals support a role for an environmental trigger(s) [57]. Associations have linked disease prevalence to reservoir source for drinking water [58]; heavy mining [59]; proximity to toxic “superfund” landfill sites [60] and oestrogen replacement therapy [61]. More recently, high levels of M8OI were found in soils in close proximity to a landfill site [19]. Since M8OI is metabolised in human liver to a metabolite that may be incorporated into lipoyl domains of autoreactive mitochondrial proteins in place of the lipoic acid [19], M8OI or related methylimidazolium ionic liquids may also be added to the list of potential triggers for PBC.

From the current study, there is little evidence to suggest that a direct interaction of M8OI with the liver could lead to hepatic changes that might lead to a development of a PBC-like disease in mice. However, M8OI (and BMI) exposure both lead to changes in GI microbiota. To date, there has been limited analyses completed on PBC patients and GI microbiota. The majority of the studies suggest that there is an association between PBC and changes in the microbiota. Lv and colleagues [62] compared 242 early stage PBC patients with 30 healthy controls and report reduced abundance of 4 species (including *Lachnobacterium spp*) and increases in 13 species in PBC patients. A more recent study [63] also reported a reduction in 4 species in 60 ursodeoxycholic acid treatment-naïve patients versus 80 matched healthy controls. Ursodeoxycholic acid is a bile acid and first line treatment for PBC [64]. An examination of microbiota changes prior to its use may be an important refinement in studies since bile acid changes themselves are likely to modulate the GI microbiota [65]. However, in terms of reduced levels, this latter study shares only 1 species in common with that by Lv *et al* [62], that of a B*acterioidetes*, with 3 shared species (*Enterobacteriacae*, *Veillonella* and *Klebsiella*) out of a total of 8 species increased. In a separate study with 39 PBC patients, significant increases in *Veillonella* (and *Eubacterium*) compared to healthy controls were reported [66]. Chen and colleagues [67] also report increases in *Veillonella* in PBC patients ([66] treatment-naïve PBC patients versus 109 healthy controls). These observations are simple associations and lack evidence for cause and effect. Despite such findings and the abundance of *Veillonella* in the adult GI-tract [68,69], there were no changes in V*eillonella* relative abundance observed in this study. This may be due the use of murine rather than human subjects. Murine and human gut microbiota do not perfectly overlap [70]. Indeed, as a genus, *Veillonella* accounted for less than 0.001% of all sequence reads obtained in this experiment. A further influence on compositional differences between this study and those by Tang [63], Abe [66] and Chen [67] *et al*., is the material from which bacteria were isolated. While previous studies have utilised stool, this study utilised caecal contents.

Comparing NOD.c3c4 mice, which develop spontaneous biliary inflammation in extra- and intrahepatic bile ducts, to control NOD mice, changes in *Veillonella* are also not flagged whereas there are changes in the levels of several other genera [71]. Interestingly, there are similarities in some of the patterns of microbiota change seen between the NOD.c3c4 and control mice and the control and ionic liquid-treated mice in this study. Most notably the second and third most differentially abundant genera associated with M8OI exposure in our study were *Dorea* and an unclassified *Clostridia*. The same two genera remained significantly enriched once cage effects were discounted in the study by Schrumpf and colleagues [71]. Further work to identify if these genera are associated with onset of biliary inflammation through immune signalling or as a result of biliary inflammation due to increased availability of bile conjugates as substrate is required.

Ionic liquids are used in multiple industrial processes and generally considered a “green” alternative to inorganic solvents [72]. Despite, multiple studies exploring the toxicity of ionic liquids on the environment, including in the contexts of plants [9], and aquatic creatures [73], none have studied the impacts of these compounds on the microbiota.

Peric *et al*. (2014), described the toxicity of both protic and aprotic ionic liquids, including BMI, on an uncharacterised soil microbiota [74]. Results of this study showed toxicity of ionic liquids (measured via respiration rates), at concentrations of 100 mg / kg and above. The authors also observed increased respiration rates of soil microbiota exposed to lower concentrations of protic ionic liquids derived from organic amines, suggesting degradation of the ionic liquids. Interestingly, they did not observe the same phenomenon in aprotic ionic liquids such as BMI and M8OI. In contrast, our results show higher proportional abundances of KEGG pathways associated with xenobiotic metabolism in mice exposed to M8OI, suggesting some degree of degradation may occur. These results are in agreement with those of past studies [75], which proposed certain microbes may utilise ionic liquids as a carbon source. Using Biolog-ECO plate methods Guo and colleagues observed enhanced growth of soil associated microbes when exposed to ionic liquids coupled with both greater amino acid and depleted phenol utilisation.

This study examined the impact of ionic liquids on mouse gut microbiota, which is compositionally distinct both taxonomically and functionally, to that of soil. Nevertheless, we build on the previous research by identifying bacterial genera proportionally enriched during ionic liquid exposure that may be responsible for degradation of these compounds in vivo. The bacterial genera enriched in ionic liquid exposed mice in this study, including *Coriobacteriaceae* and *Ruminococcus*, were also enriched in studies investigating environmental stress [43]. These same genera have also been associated with high fat diets and inflammatory response [75]. Further research to identify impact of environmental exposure to ionic liquids on immune responses and metabolism would be pertinent.

There remain some limitations to this study. Ionic liquids are not on the list of chemicals routinely screened by the EPA [https://www.epa.gov/assessing-and-managing-chemicals-under-tsca/sunset-dates-chemicals-subject-final-tsca-section-4-test]; a UK EPA equivalents (SEPA/EA) [https://www.sepa.org.uk/media/59968/policy_61-control-of-priority-and-dangerous-substances-and-specific-pollutants-in-the-water-environment.pdf] [https://www.gov.uk/government/publications/list-of-chemicals-for-water-framework-directive-assessments]; the US CDC national biomonitoring program [https://www.cdc.gov/biomonitoring/environmental_chemicals.html]; the EU water framework priority pollutant list [https://ec.europa.eu/environment/water/water-framework/priority_substances.htm] or on the priority list of the Human Biomonitoring in Europe initiative [https://www.hbm4eu.eu]. Accordingly, it is not possible to determine the degree to which the environment might be contaminated with ionic liquids and therefore to make some estimation of likely population exposure or the appropriate dose(s) to use in experimental studies that reflect likely human exposure.

There will also always be uncertainty regarding the translation of findings from experimental animals to man. The only solution to this concern is to perform similar experiments in man, but such experiments are un-ethical. However, the novel observation reported in our manuscript is that changes in microbiota occur in mice on exposure to 2 different methylimidazolium ionic liquids in the absence of any overt toxic effects to the host. This observation can reasonably be translated to man in the sense that changes likely would occur in man exposed similarly to methylimidazolium ionic liquids. Given the differences in microbiotas between mouse and man, the relative changes in species will likely be different.

An additional limitation in this study is the absence of an assessment of any intestinal effects, such as changes in mucus thickness and/or barrier function. Intestinal tissues in this study were not retained for pathology, in part, because the gut contents were manually squeezed from the tissue (which could have contributed to artefactual tissue changes associated with this procedure, rather than due solely to the ionic liquid exposures). Effects of ionic liquids on intestinal barrier function remains undetermined and should be examined in the future.

## Conclusions

While this study shows limited impact of ionic liquid exposure on host organs such as the liver and kidneys, we demonstrate taxonomic shifts in gut microbiota and suggest altered bacterial function repertoires based on predicted metagenomes. Frequent links have been made between altered gut microbiota composition and host health. Due to the interaction between the microbiota and host, these results highlight the need for future research to explore the impact of ionic liquids in humans and human associated microbiota.

## Supporting information

S1 Fig Prediction of passive absorption and bioavailabilities of 5 methylimidazolium ionic liquids. The oral absorptions and bioavailabilities of 5 structurally-related methylimidazolium ionic liquids–including BMI and M8OI–where predicted using the ACD/Percepta software as described (http://perceptahelp.acdlabs.com/help_v2017/index.php/Absorption; http://perceptahelp.acdlabs.com/help_v2017/index.php/Oral_Bioavailability) and freely accessible here: https://www.psds.ac.uk/. Based on passive absorption alone, significant (>20%) amounts of ionic liquid were predicted to be absorbed with BMI predicted to show greater maximum passive absorption compared to M8OI. In terms of bioavailability, M8OI was predicted to have low bioavailability, which was supported by the low levels of serum M8OI determined (Fig 3B). In contrast, BMI was predicted to be significantly more bioavailable than M8OI.
(PPTX)

S2 Fig Sample sizes of samples used in the determination of gut microbial composition. Sequences per sample for experimental samples (M8OI = blue; BMI = orange; Control = green) compared to negative controls (sequencing and kit negatives = black). Also included is an additional control, 2 samples of the buffer used to store stools during storage and transport (purple).
(PPTX)

S3 Fig Compositional abundance of gut microbial samples. **A**, total count compositional abundance (y axis) versus sample identity. Control communities in left panel were significantly distinct from samples in right panel (p(adj) <0.005 [pairwise PERMANOVA]). **B**, total count compositional abundance (y axis) versus sample identity after removing the main contributing OTU in the control community (Escherichia/Shigella [pale green in (A)]) prior to subsequent analysis.
(PPTX)

S4 Fig S4 Fig linkage clustering. **A**, clusters as defined by complete linkage clustering (CST1 = red; CST2 = blue; CST3 = green). **B**, validation by gap statistic.
(PPTX)

S1 Material Overview of the terms used in microbial ecology. (DOCX)

S2 Material Raw sequence data processing. (DOCX)

S1 Raw images (TIF)

S1 Table Liver glycogen levels and serum glucose concentration in mice at time of termination. (DOCX)

S2 Table Liver histopathology scores. (DOCX)

S3 Table Kidney histopathology scores. (DOCX)

S4 Table Comparison of microbial beta diversity of gut contents from each CST. (DOCX)
