# Supplementary figures and images for: Changes in the gut microbiota of mice orally exposed to methylimidazolium ionic liquids
Source: PLoS One. 2020 Mar 12;15(3):e0229745. doi: 10.1371/journal.pone.0229745 (PMC7067480; doi:10.1371/journal.pone.0229745)

## Slide 1
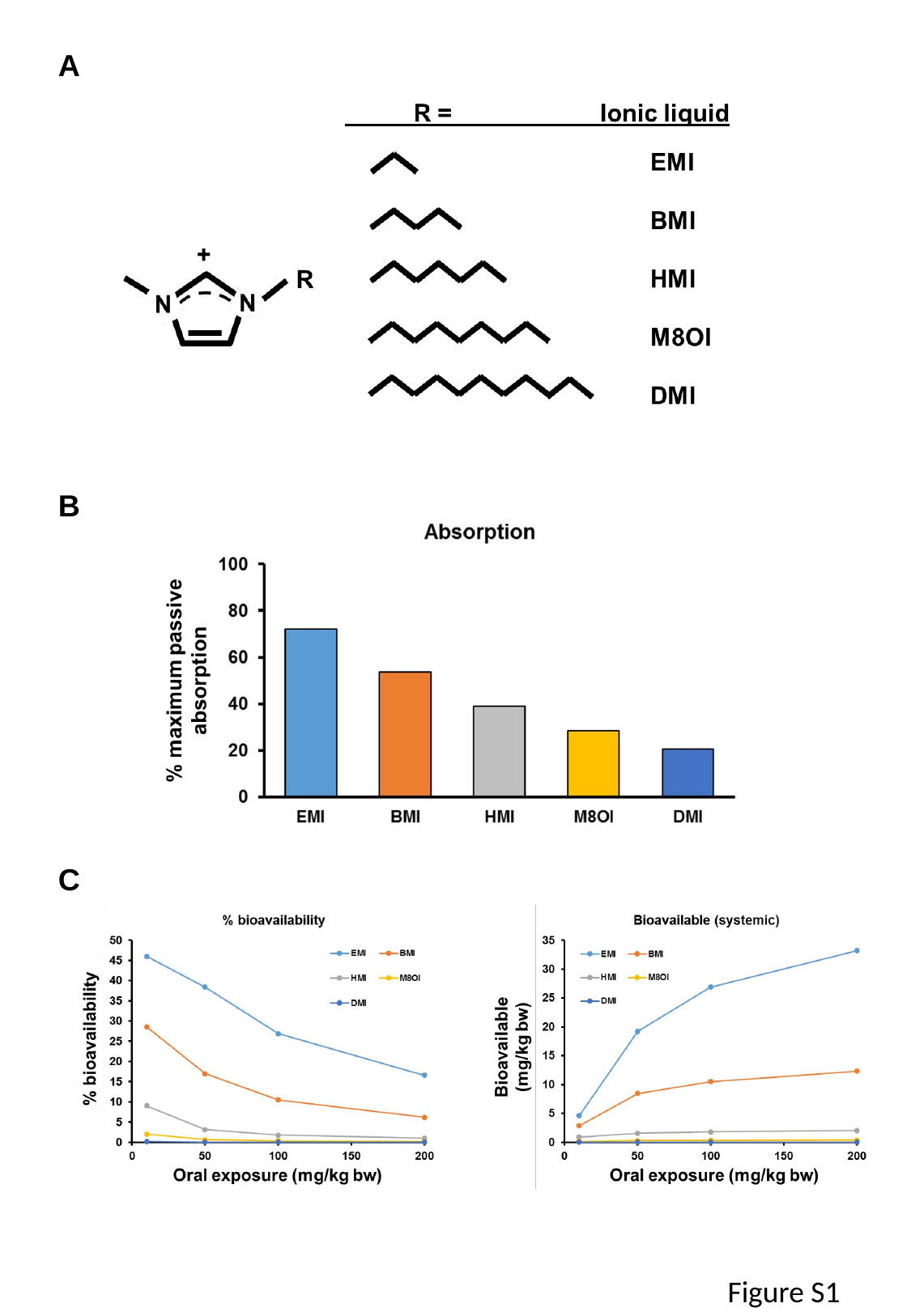

A
B
C
Figure S1

Supplement: S1 Fig — The oral absorptions and bioavailabilities of 5 structurally-related methylimidazolium ionic liquids–including BMI and M8OI–where predicted using the ACD/Percepta software as described (http://perceptahelp.acdlabs.com/help_v2017/index.php/Absorption; http://perceptahelp.acdlabs.com/help_v2017/index.php/Oral_Bioavailability) and freely accessible here: https://www.psds.ac.uk/. Based on passive absorption alone, significant (>20%) amounts of ionic liquid were predicted to be absorbed with BMI predicted to show greater maximum passive absorption compared to M8OI. In terms of bioavailability, M8OI was predicted to have low bioavailability, which was supported by the low levels of serum M8OI determined (Fig 3B). In contrast, BMI was predicted to be significantly more bioavailable than M8OI. (PPTX) [file pone.0229745.s001.pptx]

## Slide 1
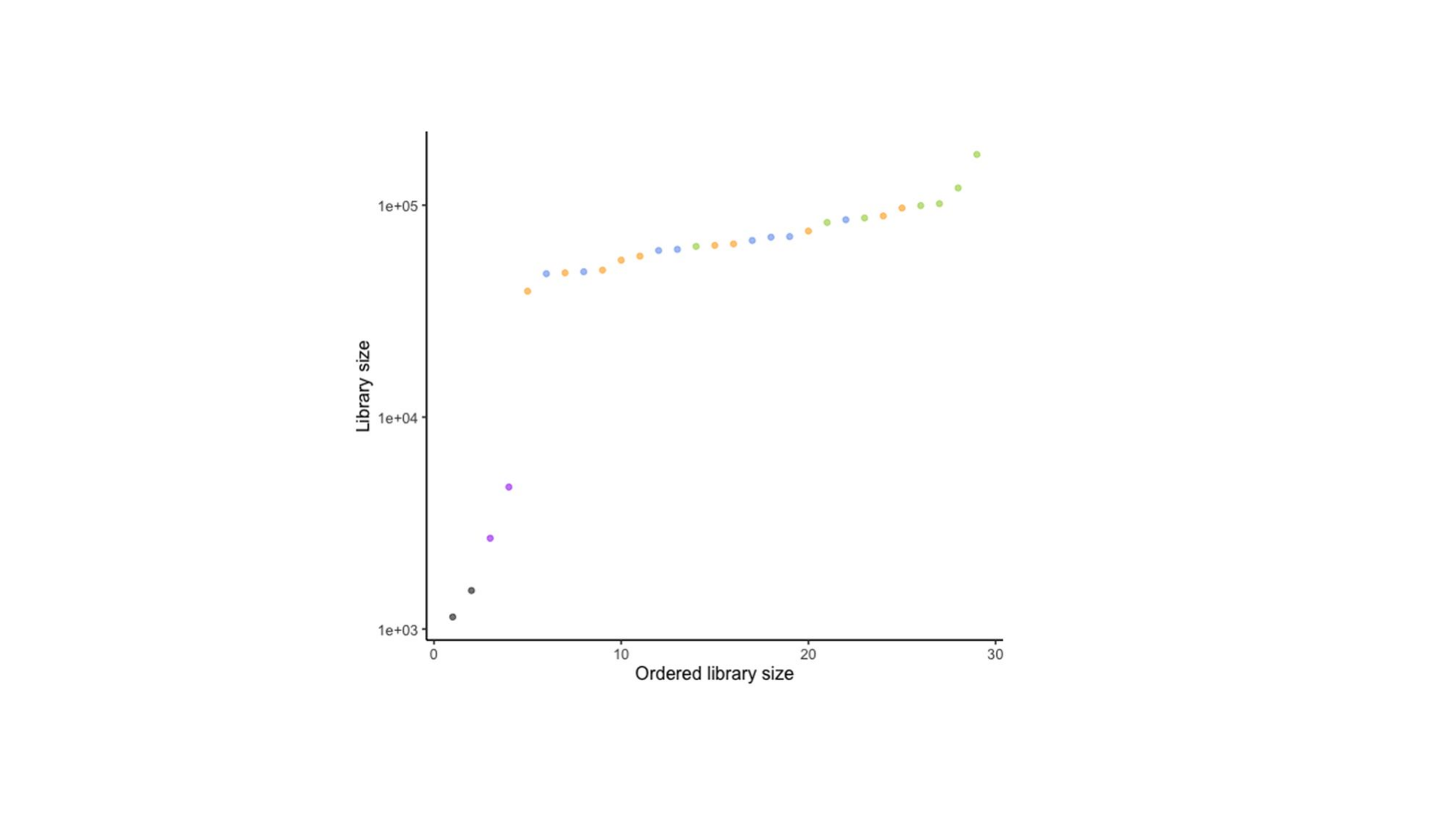

Supplement: S2 Fig — Sequences per sample for experimental samples (M8OI = blue; BMI = orange; Control = green) compared to negative controls (sequencing and kit negatives = black). Also included is an additional control, 2 samples of the buffer used to store stools during storage and transport (purple). (PPTX) [file pone.0229745.s002.pptx]

## Slide 1
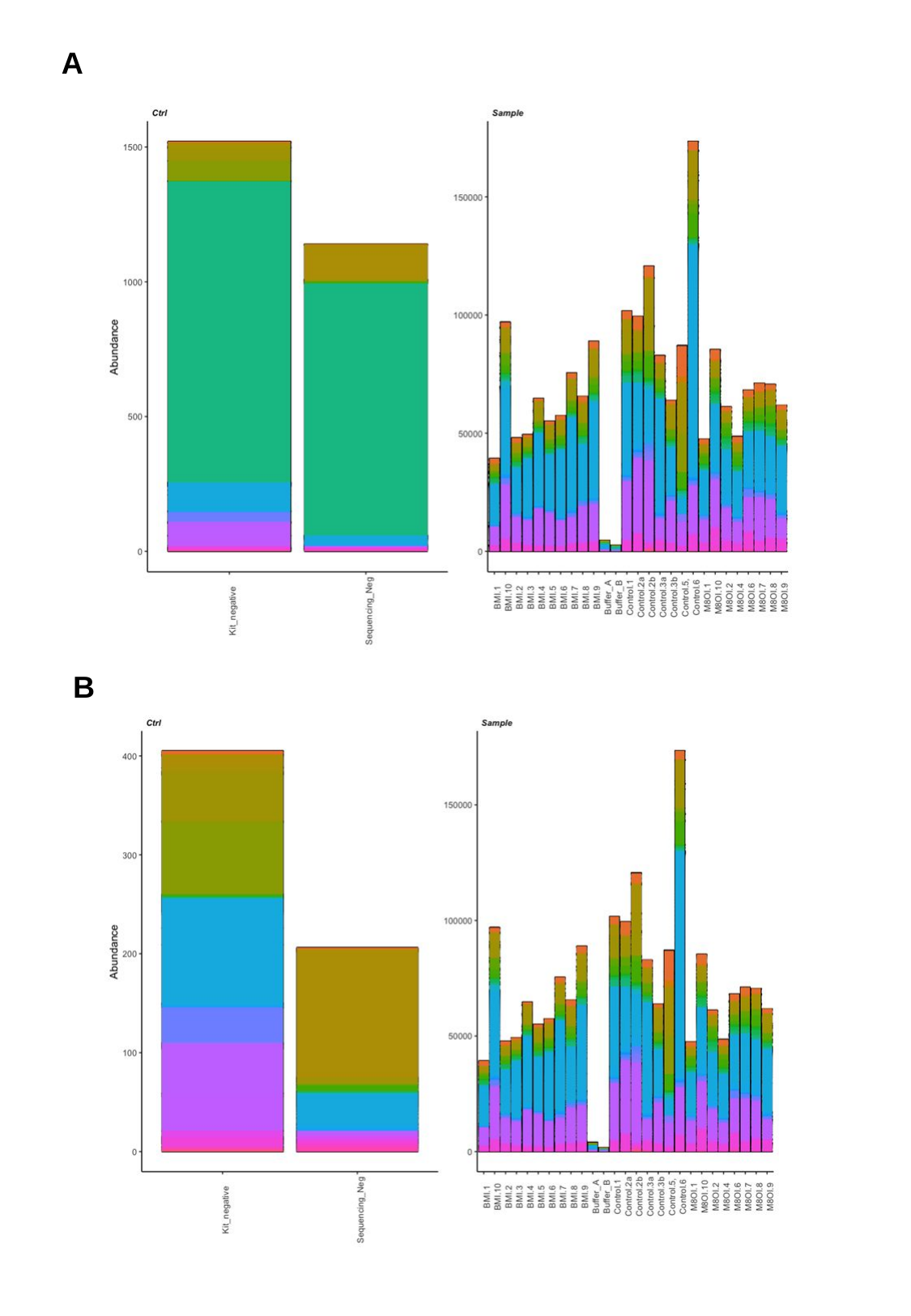

A
B

Supplement: S3 Fig — A, total count compositional abundance (y axis) versus sample identity. Control communities in left panel were significantly distinct from samples in right panel (p(adj) <0.005 [pairwise PERMANOVA]). B, total count compositional abundance (y axis) versus sample identity after removing the main contributing OTU in the control community (Escherichia/Shigella [pale green in (A)]) prior to subsequent analysis. (PPTX) [file pone.0229745.s003.pptx]

## Slide 1
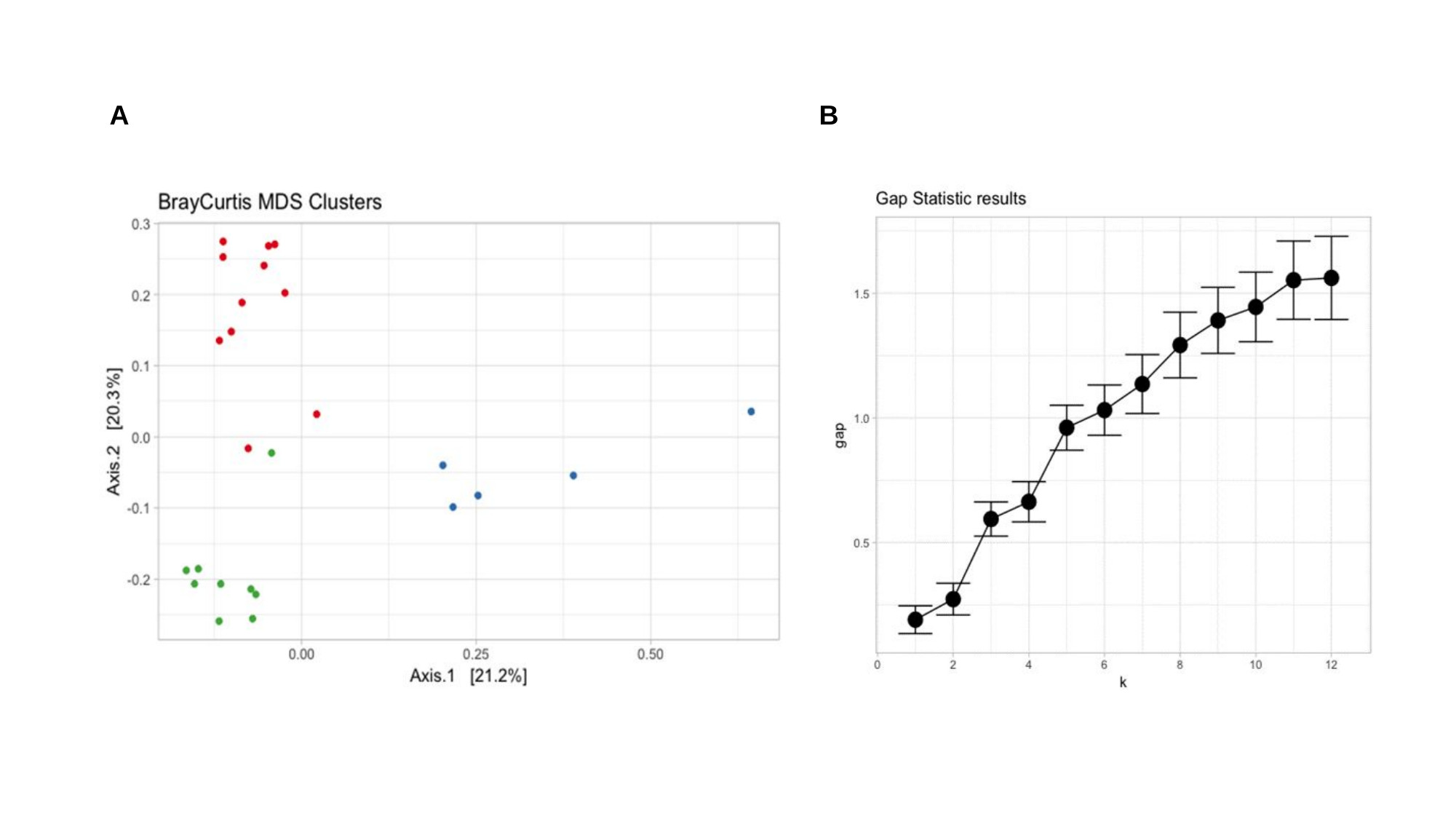

A
B

Supplement: S4 Fig — A, clusters as defined by complete linkage clustering (CST1 = red; CST2 = blue; CST3 = green). B, validation by gap statistic. (PPTX) [file pone.0229745.s004.pptx]

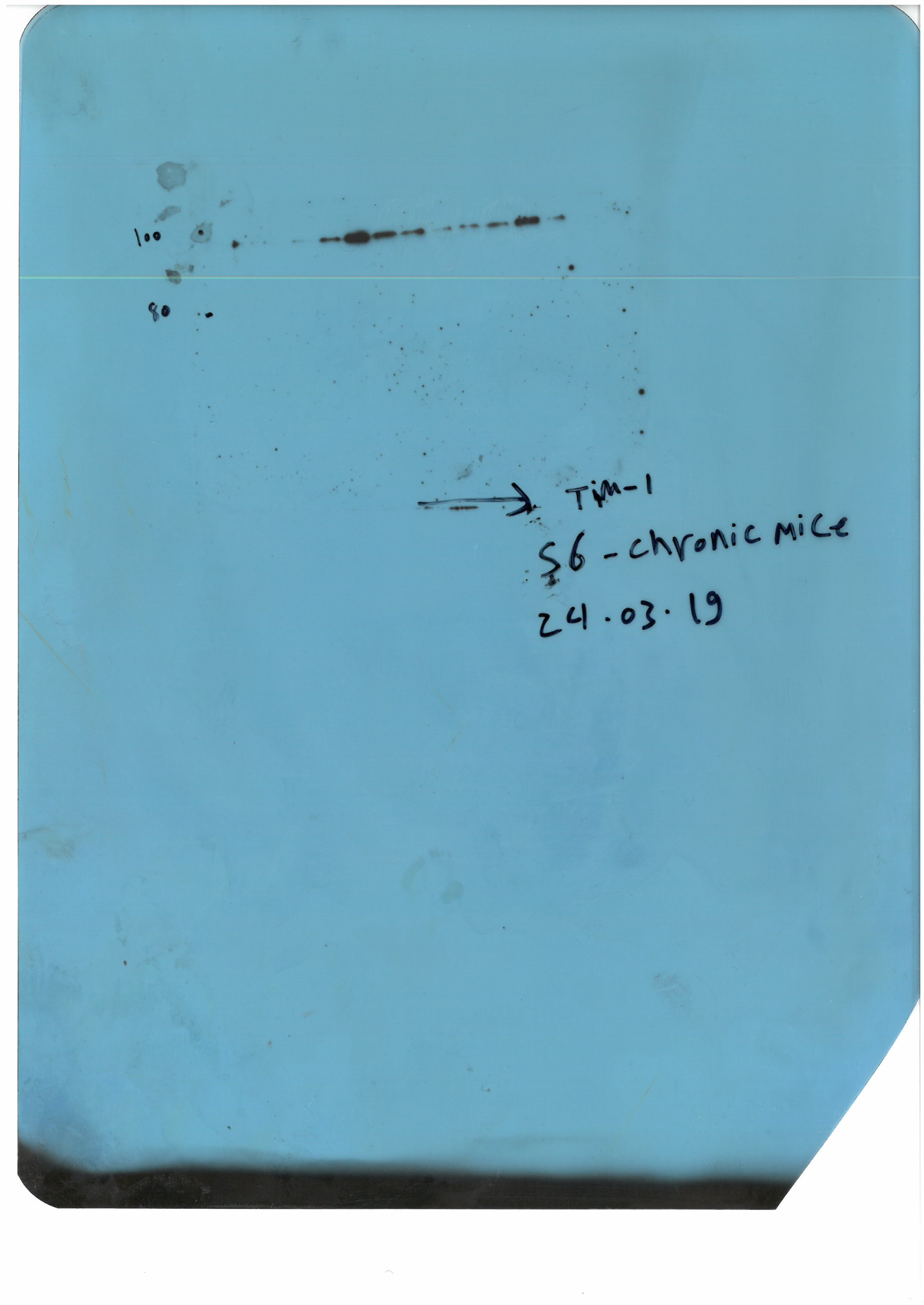

Supplement: S1 Raw images — (TIF) [file pone.0229745.s007.tif]
