# Supplementary material for: Changes in the gut microbiota of mice orally exposed to methylimidazolium ionic liquids
Source: PLoS One. 2020 Mar 12;15(3):e0229745. doi: 10.1371/journal.pone.0229745 (PMC7067480; doi:10.1371/journal.pone.0229745)
Supplement: S1 Material — (DOCX) [file pone.0229745.s005.docx]

**Supplementary Material S1. Overview of the terms used in microbial ecology.**

| Term | Definition |
| --- | --- |
| 16S rRNA gene | A bacterial gene coding for the 16S subunit of the prokaryotic ribosome. Used for microbiota analysis because presence of hyper-variable regions, flanked by conserved regions enables universal binding of primers to amplicons of highly discriminative sequences |
| Targeted sequencing | Experimental protocol deliberately aiming to identify variants of specific DNA sequence. Often target variable regions of well conserved genes to infer phylogeny or taxonomic composition. This experiment targets specifically the V4 variable region of the 16S rRNA gene. |
| Reads | A measure of sequence “hits” or number of times a sequence is identified in the data set. |
| Microbiota | The collection of microbes living within a defined environment (who is there), used interchangeably with community. |
| OTU (Operational taxonomic unit) | Group of sequences clustered together at specified similarity level (97% in this case), and assigned taxonomy based on similarity to sequence database. |
| Negative control | “Blank” sample (DNA free water) loaded on sequencer along with samples to determine level of contamination within dataset. |
| Kit control | “Blank” kit extraction (just kit reagents) performed alongside DNA extraction protocol to identify contamination in kit reagents. |
| Alpha diversity | Combined measure of richness (number of different species), and evenness (spread of different species), within a bacterial community. Calculated in this study as Fisher Alpha diversity. |
| Beta diversity | Measure of community dissimilarity between two samples. Comparing within group beta diversity to between group beta diversity enables identification of real differences between groups. Calculated in this study as Bray-Curtis diversity |
| Dominance | The opposite of evenness. A measure of abundance distribution between bacteria within a sample. Calculated in this study as Inverse Simpson diversity |
| CST (Community state type) | Groups of samples clustered together based on calculated beta diversity and validated by assessing overlap between clusters. |
| Predicted metagenome | Uses taxonomic composition to estimate the functional capacity of a community. Predicted metagenomes align taxonomic assignment with whole genome sequences deposited in databases to identify potential function of the bacteria identified during targeted sequencing experiments. |
