# Supplementary material for: Changes in the gut microbiota of mice orally exposed to methylimidazolium ionic liquids
Source: PLoS One. 2020 Mar 12;15(3):e0229745. doi: 10.1371/journal.pone.0229745 (PMC7067480; doi:10.1371/journal.pone.0229745)
Supplement: S2 Material — (DOCX) [file pone.0229745.s006.docx]

**Supplementary Material S2. Raw sequence data processing.**

Total samples - 29 *(inlcuding 2 x buffer, 1 x kit and 1 x sequencing control)*

All fastq files were processed in Mothur using commands outlined in italics below with parameters described where appropriate:

Forward and reverse reads merged *(‘make.contigs’)*

Total raw sequence reads - 2641010 *(‘summary.seqs’)*

Total sequences passing filter - 2142531 *(‘screen.seqs’maxambig=0, maxlength=275)*

Total unique seqs - 163289 *(‘unique.seqs’)*

Aligned sequences to db *(‘align.seqs’ using SILVA v4 fasta database)*

Removed chimeric sequences *(‘chimera.uchime’, dereplicate=T)*

Filtered total sequences - 2010155 *(‘filter.seqs’ vertical=T, trump=.; ‘remove.seqs’)*

Filtered unique sequences - 21322 *(‘summary.seqs’)*

Classified sequence taxonomy *(‘classify.seqs’ using SILVA non-redundant database v128, cutoff=80)*

Removed all non-bacterial seqs *(‘remove.lineage’, taxon=Chloroplast-Mitochondria-unknown- Archaea-Eukaryota-Bacteria;unknown)*

Re-filtered total seqs - 2010140 *(‘summary.seqs’)*

Re-filtered unique seqs - 21312

Computed dissimilarity between sequences *(‘dist.seqs’)*

Clustered sequences in to OTUs based on dissimilarity *(‘cluster’, method=opti)*

Classified OTUs *(‘classify.OTU’, label=0.03)*

Exported as .biom file *(‘make.biom’)*

Imported .biom file in to Rstudio for further analysis

Mean seqs / sample - 65292.1

SD seqs / sample - 36811.1
