# Supplementary material for: Changes in the gut microbiota of mice orally exposed to methylimidazolium ionic liquids
Source: PLoS One. 2020 Mar 12;15(3):e0229745. doi: 10.1371/journal.pone.0229745 (PMC7067480; doi:10.1371/journal.pone.0229745)
Supplement: S1 Table — (DOCX) [file pone.0229745.s008.docx]

**Table S1. Liver glycogen levels and serum glucose concentration in mice at time of termination.**

| **ENDPOINT** | **TREATMENT** | | | | |
| --- | --- | --- | --- | --- | --- |
|  | **Control** |  | **BMI** |  | **M8OI** |
|  |  |  |  |  |  |
| Glycogen content (µg/mg protein) | 5.4 +/- 0.52 |  | 4.1 +/- 0.29 |  | 3.9 +/- 0.23 |
| Serum glucose (mM) | 3.6 +/- 0.34 |  | 4.5 +/- 0.68 |  | 4.9 +/- 1.12 |
