# Supplementary material for: Changes in the gut microbiota of mice orally exposed to methylimidazolium ionic liquids
Source: PLoS One. 2020 Mar 12;15(3):e0229745. doi: 10.1371/journal.pone.0229745 (PMC7067480; doi:10.1371/journal.pone.0229745)
Supplement: S2 Table — (DOCX) [file pone.0229745.s009.docx]

**Table S2. Liver histopathology scores.**

| **Treatment group/**  **animal #** | **Glycogen depletion determined by PAS staining**  (minimal/  patchy/  extensive) | **Hypoeosinophilia**  (absent/minimal/mild/marked) | **Number of intra-lobular focus of inflammatory cells** (>10 inflammatory cells/focus) | **Number of inflamed portal tracts/section** (> 20 inflammatory cells/portal tract) |
| --- | --- | --- | --- | --- |
|  |  |  |  |  |
| **Control-1** | Minimal | Mild | 0 | 0 |
| **Control-2** | Patchy | Minimal | 2 | 7 |
| **Control-3** | Extensive | Mild | 2 | 1 |
| **Control-4** | Minimal | Minimal | 0 | 0 |
|  |  |  |  |  |
| **BMI-1** | Minimal | Minimal | 2 | 0 |
| **BMI-2** | Minimal | Minimal | 3 | 0 |
| **BMI-3** | Extensive | Mild | 0 | 0 |
| **BMI-4** | Patchy | Mild | 1 | 0 |
| **BMI-5** | Extensive | Absent | 2 | 0 |
| **BMI-6** | Patchy | Absent | 0 | 0 |
| **BMI-7** | Extensive | Minimal | 1 | 2 |
| **BMI-8** | Minimal | Minimal | 5 | 6 |
| **BMI-9** | Minimal | Minimal | 3 | 1 |
| **BMI-10** | Minimal | Absent | 1 | 0 |
|  |  |  |  |  |
| **M8OI-1** | Minimal | Minimal | 1 | 1 |
| **M8OI-2** | Patchy | Mild | 1 | 0 |
| **M8OI-3** | Extensive | Marked | 0 | 0 |
| **M8OI-4** | Extensive | Marked | 0 | 0 |
| **M8OI-5** | Patchy | Marked | 13 | 0 |
| **M8OI-6** | Extensive | Marked | 0 | 0 |
| **M8OI-7** | Patchy | Marked | 1 | 7 |
| **M8OI-8** | Extensive | Marked | 0 | 0 |
| **M8OI-9** | Minimal | Marked | 0 | 1 |
| **M8OI-10** | Extensive | Marked | 0 | 1 |
