# Supplementary material for: Changes in the gut microbiota of mice orally exposed to methylimidazolium ionic liquids
Source: PLoS One. 2020 Mar 12;15(3):e0229745. doi: 10.1371/journal.pone.0229745 (PMC7067480; doi:10.1371/journal.pone.0229745)
Supplement: S3 Table — (DOCX) [file pone.0229745.s010.docx]

**Table S3. Kidney histopathology scores.**

| **Treatment group/ animal #** | **Glomeruli changes**  (absent/minimal/mild/marked) | **Tubular hydropic degeneration and/or desquamation** **changes** (absent/minimal/mild/marked) |
| --- | --- | --- |
|  |  |  |
| **Control-1** | Absent | Absent |
| **Control -2** | Absent | Absent |
| **Control-3** | Absent | Absent |
| **Control-4** | Minimal | Absent |
|  |  |  |
| **BMI-1** | Marked | Marked |
| **BMI-2** | Mild | Mild |
| **BMI-3** | Mild | Absent |
| **BMI-4** | Marked | Marked |
| **BMI-5** | Mild | Absent |
| **BMI-6** | Marked | Marked |
| **BMI-7** | Mild | Mild |
| **BMI-8** | Absent | Absent |
| **BMI-9** | Mild | Marked |
| **BMI-10** | Mild | Marked |
|  |  |  |
| **M8OI-1** | Mild | Mild |
| **M8OI-2** | Mild | Marked |
| **M8OI-3** | Mild | Marked |
| **M8OI-4** | Absent | Absent |
| **M8OI-5** | Mild | Mild |
| **M8OI-6** | Absent | Absent |
| **M8OI-7** | Absent | Absent |
| **M8OI-8** | Mild | Absent |
| **M8OI-9** | Absent | Absent |
| **M8OI-10** | Mild | Mild |
