# Supplementary material for: Changes in the gut microbiota of mice orally exposed to methylimidazolium ionic liquids
Source: PLoS One. 2020 Mar 12;15(3):e0229745. doi: 10.1371/journal.pone.0229745 (PMC7067480; doi:10.1371/journal.pone.0229745)
Supplement: S4 Table — (DOCX) [file pone.0229745.s011.docx]

**Table S4. Comparison of microbial beta diversity of gut contents from each CST.**

|  | **CST 1** | **CST 2** |
| --- | --- | --- |
| **CST 1** | - | 0.003 |
| **CST 3** | 0.003 | 0.006 |

All comparisons proved significantly different beta diversities (Bray-Curtis) between pairwise CST comparisons (pairwise PERMANOVA with Bonferroni correction).

CST = Community state type
